# Vitamin A regulates mitochondrial biogenesis and function through p38 MAPK-PGC-1α signaling pathway and alters the muscle fiber composition of sheep

**DOI:** 10.1186/s40104-023-00968-4

**Published:** 2024-02-04

**Authors:** Pengkang Song, Jiamin Zhao, Fanqinyu Li, Xiaoyi Zhao, Jinxin Feng, Yuan Su, Bo Wang, Junxing Zhao

**Affiliations:** 1 College of Animal Science, Shanxi Agricultural University, Taigu, Shanxi 030801 People’s Republic of China; 2 State Key Laboratory of Animal Nutrition and Feeding, Department of Animal Nutrition and Feed Science, College of Animal Science and Technology, China Agricultural University, Beijing, 100193 People’s Republic of China

**Keywords:** Mitochondria, Muscle fiber type, PGC-1α, p38 MAPK, Retinoic acid, Vitamin A

## Abstract

**Background:**

Vitamin A (VA) and its metabolite, retinoic acid (RA), are of great interest for their wide range of physiological functions. However, the regulatory contribution of VA to mitochondrial and muscle fiber composition in sheep has not been reported.

**Method:**

Lambs were injected with 0 (control) or 7,500 IU VA palmitate into the biceps femoris muscle on d 2 after birth. At the age of 3 and 32 weeks, *longissimus dorsi* (LD) muscle samples were obtained to explore the effect of VA on myofiber type composition. In vitro, we investigated the effects of RA on myofiber type composition and intrinsic mechanisms.

**Results:**

The proportion of type I myofiber was greatly increased in VA-treated sheep in LD muscle at harvest. VA greatly promoted mitochondrial biogenesis and function in LD muscle of sheep. Further exploration revealed that VA elevated PGC-1α mRNA and protein contents, and enhanced the level of p38 MAPK phosphorylation in LD muscle of sheep. In addition, the number of type I myofibers with RA treatment was significantly increased, and type IIx myofibers was significantly decreased in primary myoblasts. Consistent with in vivo experiment, RA significantly improved mitochondrial biogenesis and function in primary myoblasts of sheep. We then used si-PGC-1α to inhibit *PGC-1α* expression and found that si-PGC-1α significantly abrogated RA-induced the formation of type I myofibers, mitochondrial biogenesis, MitoTracker staining intensity, UQCRC1 and ATP5A1 expression, SDH activity, and enhanced the level of type IIx muscle fibers. These data suggested that RA improved mitochondrial biogenesis and function by promoting PGC-1α expression, and increased type I myofibers. In order to prove that the effect of RA on the level of PGC-1α is caused by p38 MAPK signaling, we inhibited the p38 MAPK signaling using a p38 MAPK inhibitor, which significantly reduced RA-induced PGC-1α and MyHC I levels.

**Conclusion:**

VA promoted PGC-1α expression through the p38 MAPK signaling pathway, improved mitochondrial biogenesis, and altered the composition of muscle fiber type.

**Graphical Abstract:**

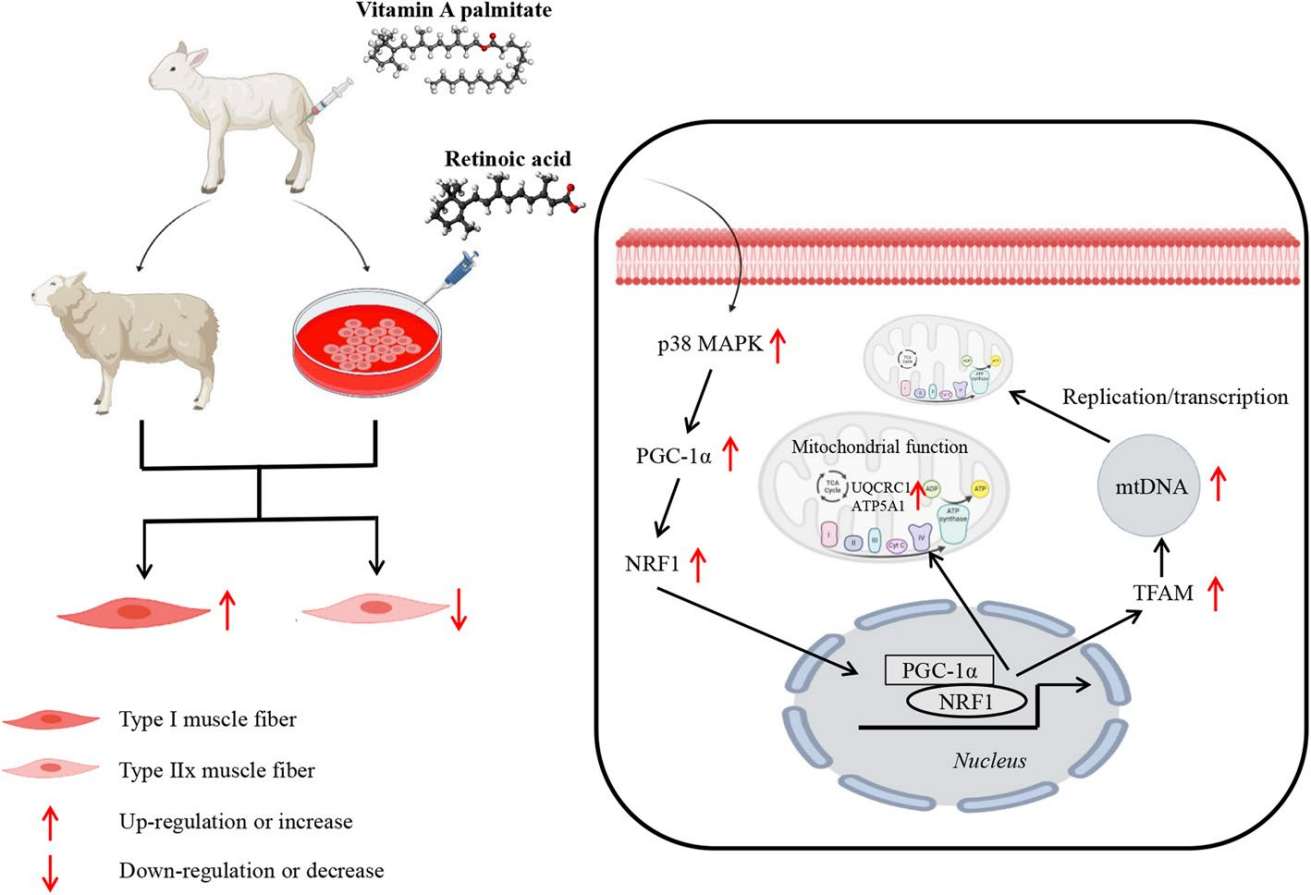

**Supplementary Information:**

The online version contains supplementary material available at 10.1186/s40104-023-00968-4.

## Background

Skeletal muscle development originates from the specification and differentiation of myoblasts and further differentiates into mature muscle fibers [1]. Muscle fiber type has been established in embryo and remodeled in adulthood through the influence of neurological, hormonal, and other factors [2]. Mature muscle fibers are composed of two myofiber types: type I and type II myofibers [3]. Moreover, myofibers are further classified into 4 types according to myosin heavy chain (MyHC) isoforms: MyHCI, MyHCIIa, MyHCIIx, and MyHCIIb [4]. Types I and IIa myofibers produce large amounts of ATP mainly through aerobic metabolic pathways, whereas types IIb and IIx myofibers rely on anaerobic glycolysis to produce a small amount of ATP [5]. In recent years, it has been shown that the addition of amino acids [6], vitamins [7] and other feed additives to diets improves the characteristics of myofibers in pigs. Therefore, using natural nutrients to alter myofiber types has become a research direction for researchers.

Myofiber type composition is strongly associated with meat quality. Oxidative myofibers increase meat redness and water-holding capacity, while type IIb myofibers contribute to poorer meat water-holding capacity and tenderness [8, 9]. Muscles with a high intramuscular fat content possess a large proportion of type I myofibers [10]. In addition, during the postmortem phase, glycolytic muscle fibers accumulate more lactic acid than oxidized muscle fibers, which leads to poorer meat quality [11–13]. Oxidized muscle fibers are rich in mitochondria and myoglobin [14]. As an energy provider, mitochondria are more abundant in oxidized muscle fibers [15]. Mitochondrial function and number are positively regulated by PGC-1α signaling, and consequently promote oxidative myofibers development [16, 17]. Accordingly, enhancing the proportion of oxidative myofibers in skeletal muscle can contribute to improving meat quality and human’s living standards.

Vitamin A (VA) is an essential fat-soluble vitamin for animals and performs vital physiological functions, including embryonic growth and development [18, 19], antioxidant capacity [20], disease resistance [21], and maintenance of vision [22]. VA has been reported to modulate PGC-1α signaling, which affects the composition of muscle fibers. For instance, intramuscular VA injection directly activates the *PGC-1α* promoter, up-regulates *PGC-1α*, and increases the proportion of oxidized muscle fibers in Angus cattle [23]. In mice, RA stimulates muscle oxidation by activating *PGC-1α* [24], and RA increases the expression of *PGC-1α* in brown adipocytes [25]. In addition, the *PGC-1α* transcriptional activity can be modulated by p38 mitogen-activated protein kinase, a post-translational modification pathway [26–28]. It has been shown that activation of the p38 MAPK signaling pathway contributes to enhance *PGC-1α* level and promote mitochondrial biogenesis [29]. It has been shown that RA increases p38 MAPK phosphorylation level and improves its function [30, 31]. Although several studies have revealed that VA and its metabolite (retinoic acid) affect myofiber type composition, whether VA affects the development of myofiber type in sheep is still unknown. In the present experiment, we hypothesized that retinoic acid may enhance *PGC-1α* expression by activating the p38 MAPK signaling pathway, promoting mitochondrial biogenesis and function, ultimately leading to an increase in oxidative muscle fibers of sheep.

## Materials and methods

### Animal ethics

This study was executed under the approval of the Institutional Animal Care and Use Committee of Shanxi Agricultural University (sxnd202028).

### Animal treatment and sampling

A total of 80 purebred Hu ewes of similar body condition were randomly selected (All ewes were in similar body weight, and had been pregnant twice previously). In order to avoid the influence by the sire, all ewes were artificially inseminated with semen from one Dorper ram after simultaneous estrus, with careful operation and hygiene during this period. After ewes became pregnant, every 3 pregnant ewes were put into one ewe shed. Ewes were fed in the stalls where they were given clean water to drink, and were free to move around after feeding. The diet was formulated to comply with the National Research Council [32] requirements for the nutrition of ewes. During the experimental period, the feeding management and environment of ewes in each group were maintained at the same level and sterilized regularly to keep the ewe shed dry and hygienic. At 35 d of gestation, fetal number was determined using an ultrasound monitor. For the follow-up study, only ewes carrying 2 fetuses were used. After birth, we selected twin lambs, both male, weighing 3.5 ± 0.5 kg, and distributed them to control and vitamin A groups to ensure the same body condition of lambs (*n* = 8 in each group).

Based on previous studies [23, 33], we decided to inject 7,500 IU VA palmitate (product No. PHR1235, Sigma, Milwaukee, USA) or an equivalent volume of corn oil (product No. c8267, Sigma, Milwaukee, USA) into the biceps femoris muscle on d 2 after birth. The lambs were given weekly injections at a fixed point in time for 3 weeks, and paired with ewes for management. Lambs weaned at 12 weeks of age and fed a backgrounding diet for 55 d, followed by a finishing diet with free access to clean water and salt blocks. Moreover, grass hay (peanut seedlings) was added to promote lamb growth during the finishing period. Nutrient composition of concentrate diet and grass hay have been reported in another manuscript [34]. For peanut seedlings, the AOAC method [35] was used to determine DM content, and the Van Soest method [36] was used to determine NDF and ADF contents. The Kjeldahl and Soxhlet extraction methods were used to measure the content of crude protein and crude fat, respectively [37]. The content of total ash in samples was measured after 40 min of carbonization in a constant temperature crucible at 600 °C.

Surgical instruments, iodophor, normal saline and 75% alcohol were prepared for surgical sampling at the age of 12 weeks of lambs. The lambs were completely anesthetized with 2% lidocaine and collected muscle tissue with surgical instruments. The samples were divided into two parts: (1) the trimmed samples were frozen with isopentane and embedded in OCT; (2) the samples were put into precooled PBS for primary myoblasts separation. All animals were harvested at 32 weeks of age, and a portion of skeletal muscle tissue collected was made into frozen sections and the other portion was stored at −80 °C.

### Sheep primary myoblasts isolation, culture and treatment

Sheep primary myoblasts were isolated according to the method previously described [34]. When the cell fusion reached 100%, the differentiation medium containing RA and 2% horse serum was replaced. Cells were collected after 6 d of differentiation. Myoblasts were isolated from 6 untreated ram lambs aged 2-day-old. The isolated cells were mixed thoroughly and seeded into 6-well plates, and then the cells were treated as control and RA groups respectively (*n* = 3 in each group).

To elucidate the relationship between RA and PGC-1α in myofiber type conversion, we used PGC-1α siRNA (Genomeditech, Shanghai, China) to inhibit the expression of *PGC-1α*. When induction of differentiation was initiated, si-PGC-1α was transfected into RA-containing cells using lipofectamine 3000. Cells were collected after 6 d of differentiation. The following is the sequence information about negative control siRNA (siNC) and si-PGC-1α. siNC: the sense strand was 5′-UUCUCCGAACGUGUCACGUtt-3′, and the antisense strand was 5′-ACGUGACACGUUCGGAGAAtt-3′. Si-PGC-1α: the sense strand was 5′-GCCAAACCAACAACUUUAUtt-3′ and the antisense strand was 5′-AUAAAGUUGUUGGUUUGGCtt-3′. To further investigate the role of p38 MAPK in the RA-PGC-1α pathway, we used the p38 MAPK inhibitor SB202190 (Solarbio, Beijing, China, Cat. No.: IS1380) to inhibit the p38 MAPK signaling pathway. SB202190 was added when induction of myoblasts differentiation was initiated, and cells were collected 6 d after differentiation.

### Enzyme activity assay

Succinate dehydrogenase (SDH, #A022-1-1) and malate dehydrogenase (MDH, #A021-2-1) activities in skeletal muscle and cells were determined according to the instructions of Nanjing Jiancheng Bioengineering Institute (Nanjing, China).

### ATPase staining

The skeletal muscle samples were embedded in optimal cutting temperature compound (OCT compound) and 8 μm sections were cut with a cryo-microtome (Leica CM1950, Wetzlar, Germany), then prepared the following two kinds of incubation solutions.

#### Alkaline dye solution

The frozen sections were dried naturally, and encircled the tissue with an immunohistochemical pen, the incubation solution of pH 10.4 was added dropwise (Tris base 5 g + calcium chloride 0.499 g + distilled water to make the volume to 250 mL, take 20 mL of it and adjust the pH to 10.4 with 0.1 mol/L hydrochloric acid) for 5 min, pour off the incubation solution, add dropwise the incubation solution with pH 9.4 (Tris base 5 g + calcium chloride 0.499 g + distilled water to dilute to 250 mL, take 20 mL of it and add 30 mg ATP sodium salt and adjust the pH to 9.4 with 0.1 mol/L hydrochloric acid) for 30 min. The incubation solution was poured out and the sections were sequentially stained with 2% calcium chloride, 2% cobalt nitrate and 1% ammonium sulfide, passed through absolute ethanol and xylene. Finally, observed and took pictures with a microscope (Leica, Wetzlar, Germany). Results: Type I muscle fibers were gray or colorless, and type II muscle fibers were dark or black.

#### Acid dye solution

Consistent with the above method, it was only necessary to replace the incubation solution of pH 10.4 with that of pH 4.6.

Results: Type I muscle fibers were dark or black, and type II muscle fibers were gray or colorless.

### Analysis of the mitochondrial DNA (mtDNA) content

Genomic DNA was extracted from LD muscle and primary myoblasts according to the M5 HiPer Universal DNA Mini Kit (Mei5 Biotechnology, Co., Ltd., Beijing, China) instruction manual. The relative content of mitochondrial DNA was determined by RT-qPCR. The primers used for mtDNA (accession number: AF039578) amplification were as follows: forward primer 5′-CGCTTGGCAAGGATCCCTCT-3′ and reverse primer 5′-CCTCAGACGGCCATAGCTGA-3′. The primers used to amplify β-actin (accession number: DQ152927) were as follows: forward primer 5′-CCGCAAATGCTTCTAGGCGG-3′ and reverse primer 5′-AACCGACTGCTGTCCCCTTC-3′.

### Transmission electron microscopy (TEM)

Primary myoblasts were grown to more than 6^th^ power of 10 and gently detached from the plates with a cell lifter. Centrifuge at 2,000 r/min for 8 min, and collect the cells in a 1.5-mL centrifuge tube. Discard the supernatant, slowly add pre-cooled 2.5% glutaraldehyde along the wall of the tube, and then fixed in a 4 °C refrigerator overnight. Fixation of samples was continued with 1% OsO_4_ in phosphate buffer (0.1 mol/L, pH = 7.0) for 1–2 h, followed by dehydration, infiltration, embedding and ultrathin sectioning. Finally, specimens were sectioned under a LEICA EM UC7 ultrasound microscope, stained sequentially with uranyl acetate and basic lead citrate for 5 to 10 min, and images were captured in a Hitachi H-7650 TEM.

### MitoTracker staining and determination

When the cells reach 80% confluence, the culture medium was removed, and the MitoTracker Red CMXRos staining solution preheated at 37 °C was added (final concentration 250 nmol/L), and incubated for 30 min under normal culture conditions. After the staining is over, the above staining solution can be replaced with fresh culture medium or PBS, which can be observed under a fluorescence microscope or read under a microplate reader (excitation wavelength 490 nm, emission wavelength 516 nm).

### Real-time quantitative PCR

Total RNA was extracted from muscle and cell using Trizo reagent (Sigma, Saint Louis, MO, USA) and synthesized into cDNA using a reverse transcription kit (Takara Co., Ltd., Dalian, China). The CFX RT-PCR detection system (Bio-Rad, Hercules, CA, USA) and SYBR Green RT-PCR kit (Takara Co., Ltd., Dalian, China) were used for q-RT-PCR. The procedure was as follows: 95 °C, 10 min; 45 2-step cycles of 95 °C, 15 s; 60 °C, 30 s, with at least 3 replicates per set. Primer sequences are shown in Table 1. The method of 2^-ΔΔCt^ was used to calculated the relative changes of gene expression, and β-actin was used as an internal reference.

**Table 1:** Primer sequences for real-time PCR

Gene name	Sequence (5′→3′)	Product size, bp
*MHC I*	F: AAGAACCTGCTGCGGCTG	250
	R: CCAAGATGTGGCACGGCT	
*MHC IIa*	F: GAGGAACAATCCAATACAAATCTATCT	173
	R: CCCATAGCATCAGGACACGA	
*MHC IIb*	F: GACAACTCCTCTCGCTTTGG	274
	R: GGACTGTGATCTCCCCTTGA	
*MHC IIx*	F: GGAGGAACAATCCAATGTCAAC	178
	R: GTCACTTTTTAGCATTTGGATGAGTTA	
*PGC-1α*	F: TATTTGCATCCAGAGCATGGC	181
	R: CCAGAGCAGCACAAAAGTACC	
*NRF1*	F: CATGGCACTCAACAGCGAAG	177
	R: GGGGTCTTCCAGAATTGGGT	
*TFAM*	F: AGGATGGCACATCACAGGTAA	168
	R: GGTCTTCTCGTCCAACTTCCA	
*UQCRC1*	F: TGGCTGGATTTGGCCCCATT	136
	R: TTTATGGTGGAGGGACGGCG	
*ATP5A1*	F: CTTCAGAAAACCGGCACTGCT	210
	R: GTCAGGCTCCAAGTTCAGAGAC	
*β-actin*	F: CGGCTTTCGGTTGAGCTGAC	159
	R: GCCGTACCCACCAGAGTGAA	

### Western blotting

Proteins from muscle and cells were extracted with RIPA lysate (1% NaF, 1% Na_3_VO_4_, 1% PMSF, 2% β-mercaptoethanol, 0.1% protease inhibitor, 1× loading buffer constant volume to 10 mL), placed at 100 °C for 10 min, then centrifuged at 4 °C, 12,000 × *g* for 8 min, and the supernatant was removed. The extracted proteins were separated by SDS-PAGE at room temperature (parameters: 80 V for 0.5 h, 120 V for 1.5 h) and then transferred proteins to nitrocellulose membranes at 4 °C (parameters: 100 V for 1.5 h) and blocked with 5% skim milk powder (Shanghai Sanger Biotechnology Co., Ltd., Shanghai, China) for 1 h. Finally, nitrocellulose membranes were incubated overnight at 4 °C with the primary antibody and 1 h at room temperature with the corresponding secondary antibody. The Odyssey Infrared Imaging System (LI-COR Biosciences, Lincoln, NE, USA) was used to visualize the protein bands and band densities were standardized to the content of β-tubulin and β-actin.

Antibodies against myosin heavy chain, Type I (BA-D5), and Myosin heavy chain, Type IIX (6H1) were from DSHB (Lowa, USA). UQCRC1 (No. 21705-1-AP) and ATP5A1 (No. 14676-1-AP) were purchased from Proteintech (Lowa, USA). Phospho-p38 MAP Kinase (Thr389, No. 9211) and p38 MAPK (No. 9212) were from Cell Signaling (Danvers, MA, USA). PGC-1α (bs1832R), β-tubulin (bsm-33034 M) and β-actin (AP0060) were purchased from Biosynthesis Biotechnology Co., Ltd. (Beijing, China). Goat anti-rabbit secondary antibody (926–32211) and anti-mouse secondary antibody (926–68070) were purchased from LI-COR Biosciences (Lincoln, NE, USA).

### Statistical analysis

Data were shown as the mean ± SEM, and analyzed using Graphpad Prism 9 software (Monrovia, CA, USA). The normal distribution and homogeneity of variance analysis were performed. The comparison of two groups of data was performed using Student’s *t*-test, and the comparison of multiple groups of data was performed using one-way analysis of variance (ANOVA) followed by Tukey’s test. *P* < 0.05 was considered to be significantly different.

## Results

### Effects of vitamin A injection on myofiber type composition in *longissimus dorsi* muscle

**Fig. 1:**
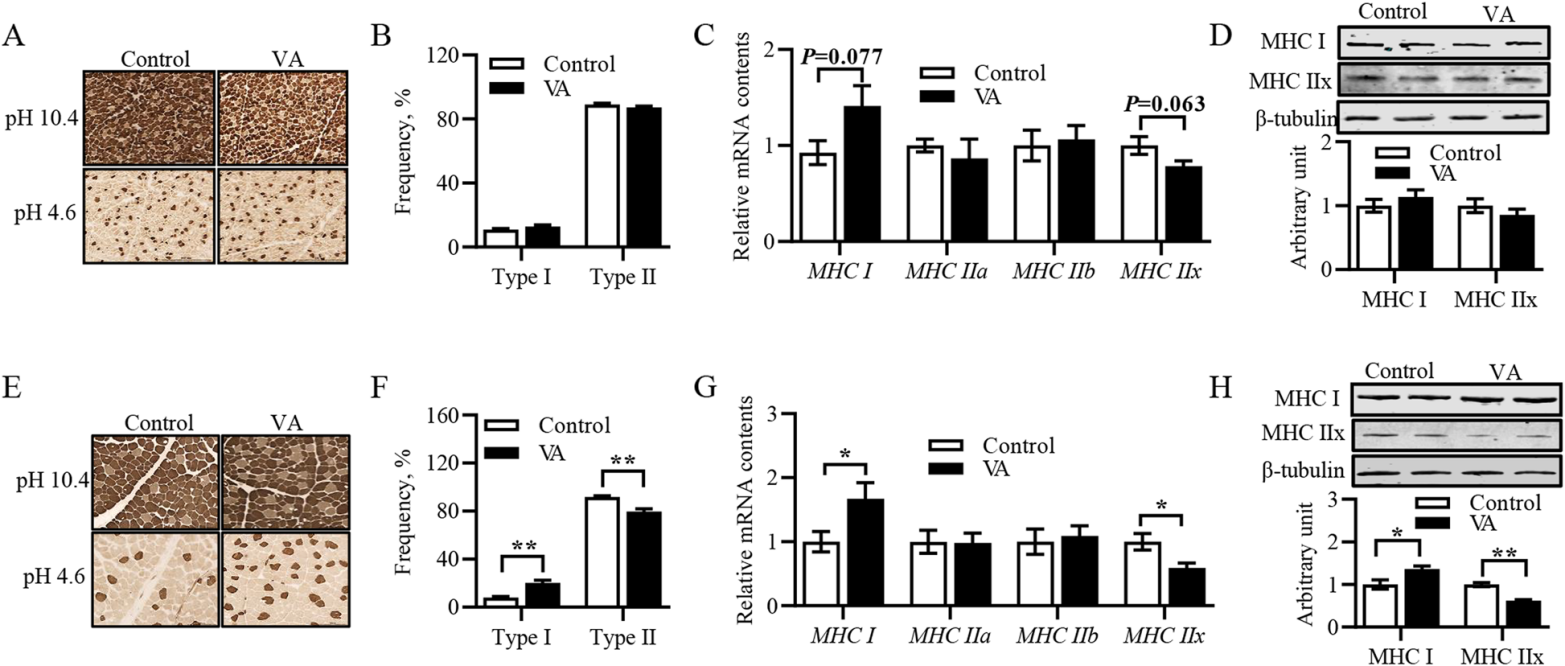
Effects of vitamin A injection on myofiber type composition in *longissimus dorsi* muscle of sheep. **A** Representative images on myofiber type composition in 3-week-old lambs. **B** The proportion of myofiber type composition in 3-week-old lambs. **C** Relative mRNA content of *MyHC I*, *MyHC IIa*, *MyHC IIb* and *MyHC IIx* in 3-week-old lambs. **D** Protein abundance of MyHC I and MyHC IIx in 3-week-old lambs. **E** Representative images on muscle fiber type composition of sheep at harvest. **F** The proportion of myofiber type composition of sheep at harvest. **G** Relative mRNA content of *MyHC I*, *MyHC IIa*, *MyHC IIb* and *MyHC IIx* of sheep at harvest. **H** Protein abundance of MyHC I and MyHC IIx of sheep at harvest. Data were shown as mean ± SEM; *n* = 8 in each group, ^*^P < 0.05, ^**^P < 0.01

Although VA did not change the muscle fiber type in LD muscle of 3-week-old lambs (Fig. 1A and B), q-RT-PCR results showed a significant increase trend in the expression of type I muscle fiber in VA group lambs (Fig. 1C, *P* = 0.077), while the expression of type IIx muscle fiber showed an opposite trend (Fig. 1C, *P* = 0.063). There were no significant changes in protein abundance of MyHC I and MyHC IIx in 3-week-old lambs (Fig. 1D). After slaughter, VA greatly increased the number of type I myofibers, decreased the number of type IIx myofibers (Fig. 1E and F). In addition, the mRNA and protein content of MyHC I was significant raised, while the mRNA and protein content of MyHC IIx was significant reduced (Fig. 1G and H).

### Vitamin A improved mitochondrial biogenesis and function, and activated p38-PGC-1α signal pathway

**Fig. 2:**
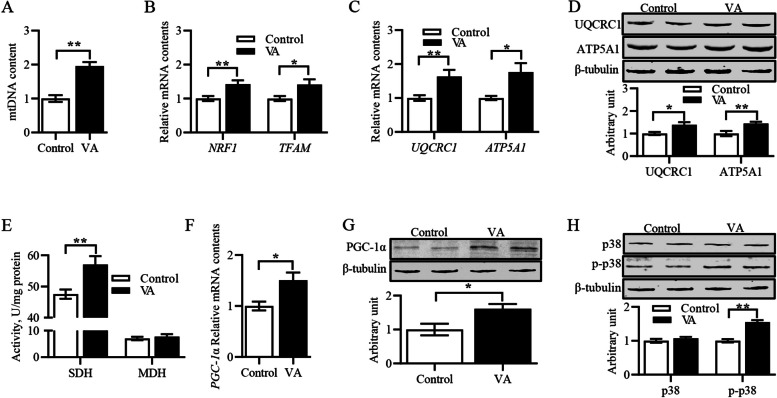
Vitamin A improved mitochondrial biogenesis and function, activated p38-PGC-1α signal pathway. **A** mtDNA content. **B** Relative mRNA content of *NRF1* and *TFAM*. **C** Relative mRNA content of *UQCRC1* and *ATP5A1*. **D** Protein expression of UQCRC1 and ATP5A1. **E** SDH and MDH activities. **F** Relative *PGC-1α* content. **G** Protein expression of PGC-1α. **H** Protein expression of p38 MAPK and p-p38 MAPK. Data were shown as mean ± SEM; *n* = 8 in each group, ^*^P < 0.05, ^**^P < 0.01

Mitochondria are the main site of aerobic respiration in cells, which is known as the “power house”, so it is necessary to know if mitochondrial biogenesis and function are regulated by VA. As shown in Fig. 2A and B, VA significantly increased mtDNA content and the mRNA levels of *TFAM* and *NRF1* in LD muscle of sheep. For mitochondrial function, VA increased UQCRC1 and ATP5A1 mRNA and protein levels, and elevated SDH activity (Fig. 2C–E). Additionally, VA elevated the PGC-1α mRNA and protein levels and enhanced p38 MAPK phosphorylation level (Fig. 2F–H). These data suggested that the improvement of mitochondrial biosynthesis and function by VA may be caused by p38 MAPK-PGC-1α signaling.

### Retinoic acid improved mitochondrial biogenesis and function, and altered myofiber type composition

**Fig. 3:**
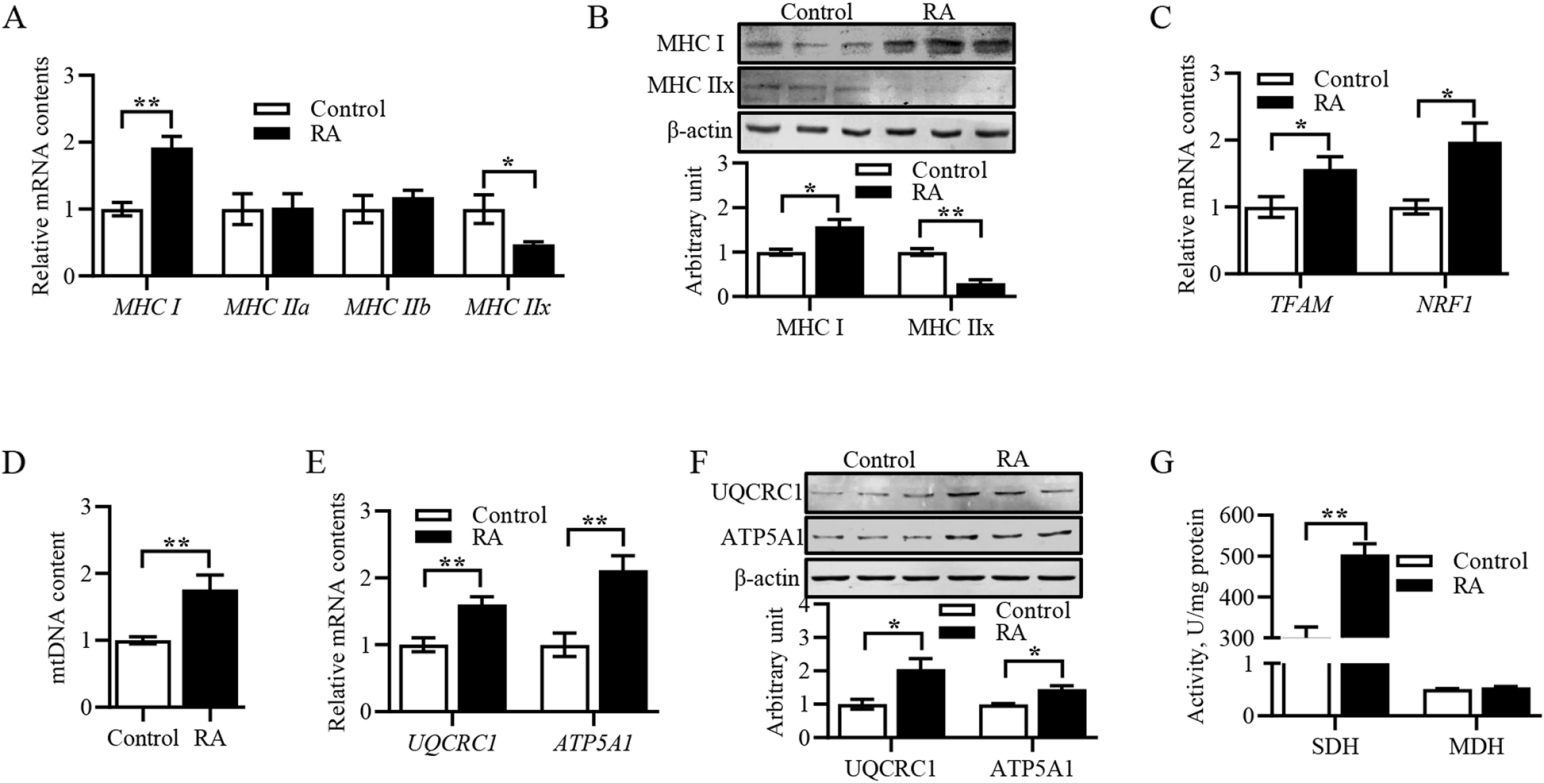
Retinoic acid improved mitochondrial biogenesis and function, altered myofiber type composition. **A** Relative mRNA content of *MyHC I*, *MyHC IIa*, *MyHC IIb* and *MyHC IIx*. **B** Protein expression of MyHC I and MyHC IIx. **C** Relative mRNA content of *TFAM* and *NRF1*. **D** mtDNA content. **E** Relative mRNA content of *UQCRC1* and *ATP5A1*. **F** Protein expression of UQCRC1 and ATP5A1. **G** SDH and MDH activities. Data were shown as mean ± SEM; *n* = 3 in each group, 3 replications were used during the experiment and each well was considered as an experiment unit from different lambs, ^*^P < 0.05, ^**^P < 0.01

We then explored whether RA exerted effects on myofiber type in primary myoblasts. In the current study, MyHC I mRNA and protein levels were significantly higher, while MyHC IIx mRNA and protein levels were significantly decreased in RA-treated primary myoblasts (Fig. 3A and B). Consistent with the experimental results in vivo, RA significantly increased *TFAM* and *NRF1* mRNA levels, mtDNA content, UQCRC1 and ATP5A1 mRNA and protein levels and SDH activity in primary myoblasts (Fig. 3C–G).

### Retinoic acid affected mitochondrial biogenesis and altered myofiber type composition through PGC-1α signaling

**Fig. 4:**
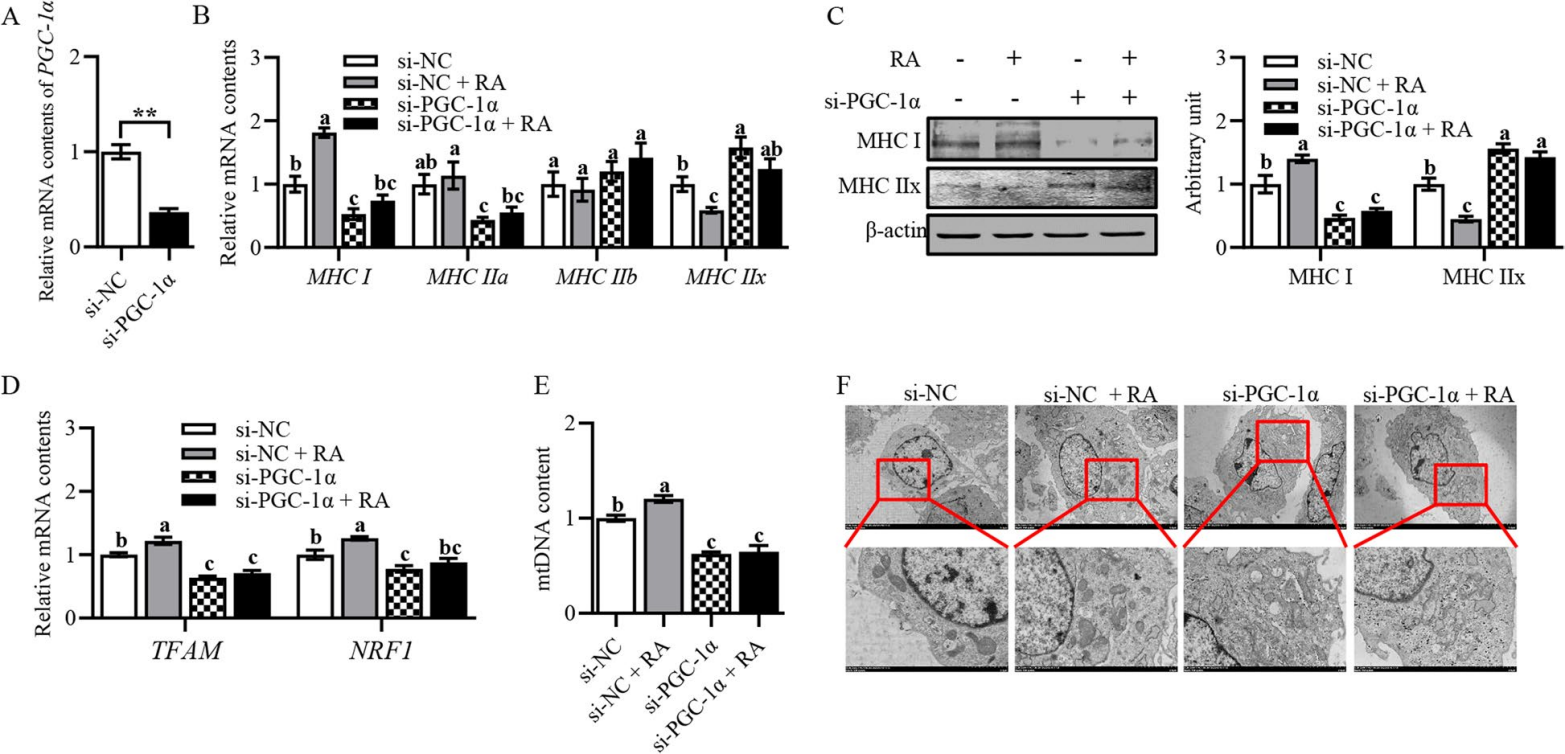
Retinoic acid affected mitochondrial biogenesis and altered myofiber type composition through PGC-1α signaling. **A** Relative mRNA content of *PGC-1α.* **B** Relative mRNA content of *MyHC I*, *MyHC IIa*, *MyHC IIb* and *MyHC IIx*. **C** Protein expression of MyHC I and MyHC IIx. **D** Relative mRNA content of *TFAM* and *NRF1*. **E** mtDNA content. **F** Representative images showing the number of mitochondria in primary myoblasts. Data were shown as mean ± SEM; *n* = 3 in each group, 3 replications were used during the experiment and each well was considered as an experiment unit from different lambs, ^**^P < 0.01. ^a^^–^^c^Values with different letters indicated significant differences (*P* < 0.05)

In order to verify whether RA affected mitochondrial biogenesis and altered muscle fiber type composition through PGC-1α signaling, we used si-PGC-1α to inhibit *PGC-1α* expression in primary myoblasts. As expected, *PGC-1α* mRNA level was significantly down-regulated (Fig. 4A). We also found type I myofiber mRNA and protein levels were significantly decreased, while type IIx myofiber mRNA and protein levels showed the opposite results (Fig. 4B and C). Furthermore, silencing of *PGC-1α* caused impaired mitochondrial biogenesis, as evidenced by a significant downregulation of *TFAM* and *NRF1* mRNA levels, and a significant reduction in mtDNA content and mitochondrial number (Fig. 4D–F). However, supplementation with RA did not alleviate these situations (Fig. 4B–F). Thus, our results suggested that RA improved mitochondrial biogenesis and altered the composition of myofibers through *PGC-1α.*

### Retinoic acid affected mitochondrial function through PGC-1α signaling

**Fig. 5:**
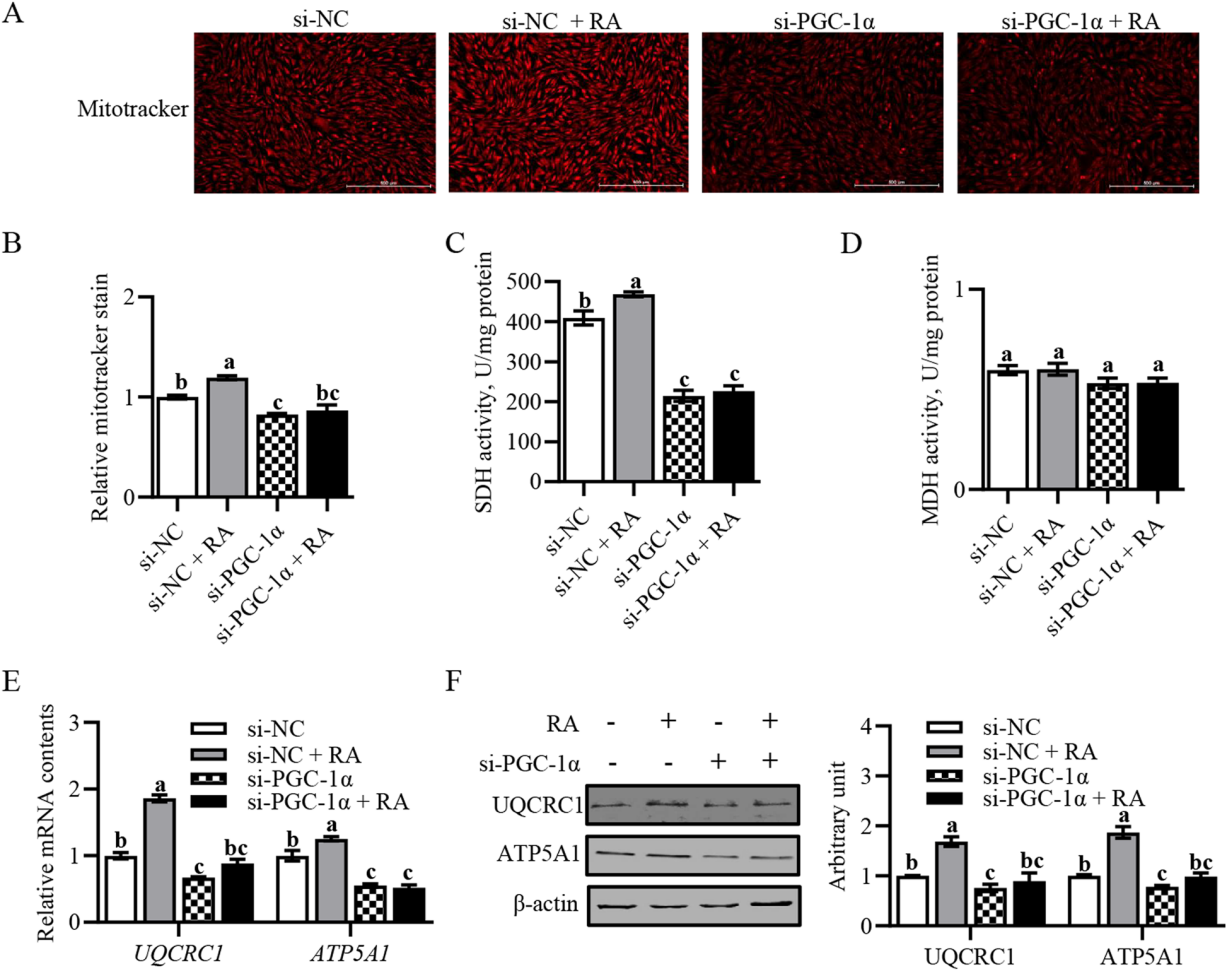
Retinoic acid affected mitochondrial function through PGC-1α signaling. **A** Representative images showing MitoTracker staining. **B** The relative intensity of MitoTracker staining. **C** SDH activity. **D** MDH activity. **E** Relative mRNA content of *UQCRC1* and *ATP5A1*. **F** Protein expression of UQCRC1 and ATP5A1. Data were shown as mean ± SEM; *n* = 3 in each group, 3 replications were used during the experiment and each well was considered as an experiment unit from different lambs. ^a^^–^^c^Values with different letters indicated significant differences (*P* < 0.05)

We further explored whether RA affected mitochondrial function through the regulation of *PGC-1α*. Consistent with our hypothesis, si-PGC-1α significantly attenuated the intensity of RA-induced MitotTracker staining (Fig. 5A and B). In addition, si-PGC-1α decreased RA-induced SDH activity, and UQCRC1 and ATP5A1 mRNA and protein levels (Fig. 5C–F). These results suggested that RA improved mitochondrial function through PGC-1α signaling.

### Retinoic acid affected myofiber type composition via p38 MAPK-PGC-1α signaling

**Fig. 6:**
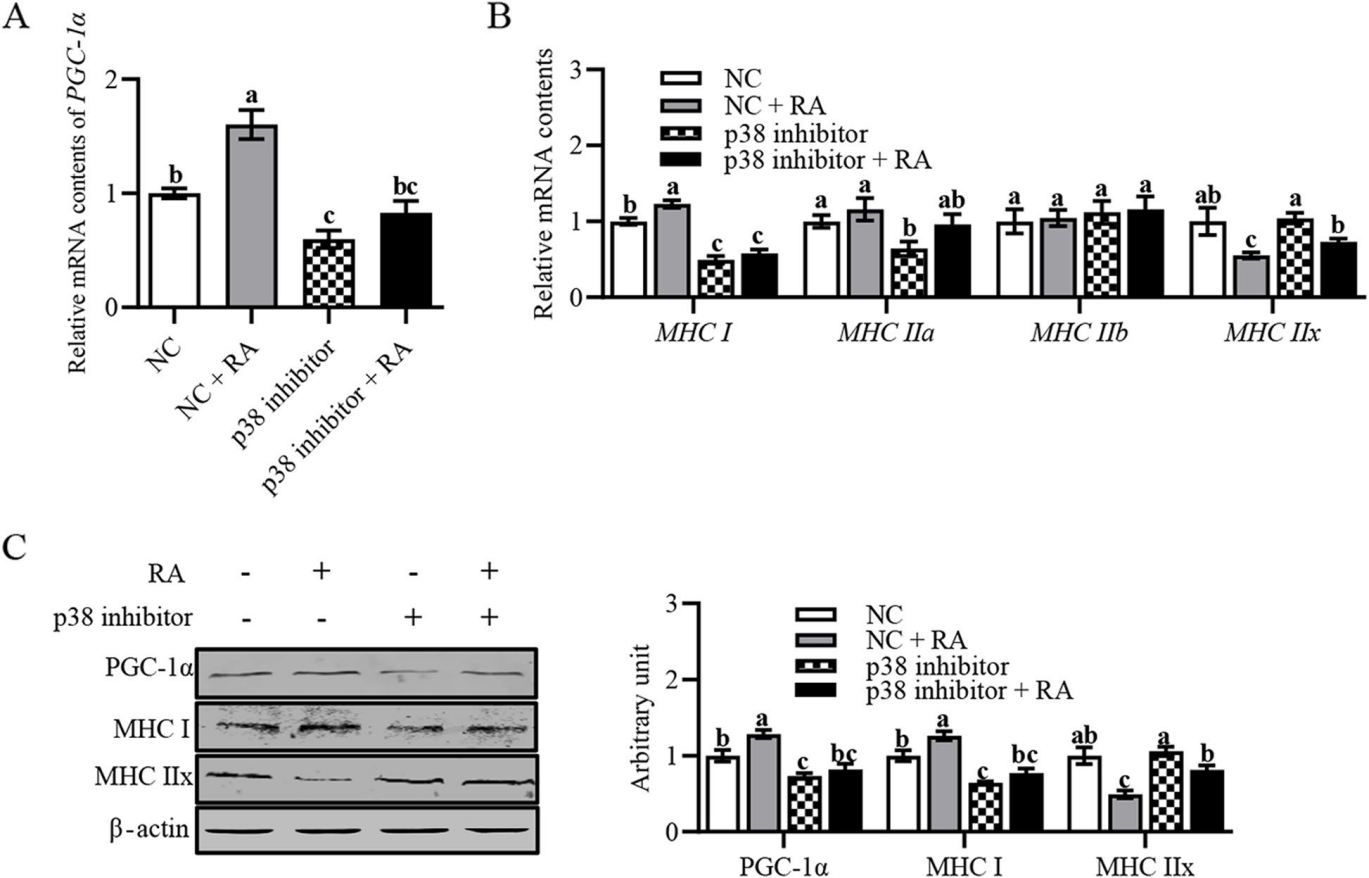
Retinoic acid affected myofiber type composition through p38 MAPK-PGC-1α signaling. **A** Relative *PGC-1α* content. **B** Relative mRNA content of *MyHC I*, *MyHC IIa*, *MyHC IIb* and *MyHC IIx*. **C** Protein expression of PGC-1α, MyHC I, and MyHC IIx. Data were shown as mean ± SEM; *n* = 3 in each group, 3 replications were used during the experiment and each well was considered as an experiment unit from different lambs. ^a^^–^^c^Values with different letters indicated significant differences (*P* < 0.05)

In order to elucidate that RA affected myofiber type composition through regulation of the p38 MAPK-PGC-1α signaling pathway, we used the p38 MAPK inhibitor (SB202190) to inhibit the p38 MAPK signaling. The results revealed that SB202190 significantly abrogated RA-induced the PGC-1α and type I myofibers mRNA and protein levels, but those of type IIx myofibers were not changed (Fig. 6A–C). These data indicate that RA significantly increased the number of oxidative myofibers via p38 MAPK-PGC-1α signaling.

## Discussion

The occurrence and conversion of different skeletal muscle fiber types are extremely important for the study of skeletal muscle development. The generation of muscle fiber types accompanies the entire process of myogenesis and is subject to complex regulation at the physiological and molecular levels. In mammals, a distinction is made between fast and slow formation of primary and secondary myofibers [38]. All primary myofibers are initially slow, with some of them subsequently converting to fast, whereas all secondary muscle fibers are initially fast, with some of them subsequently converting to slow [38]. With the number of muscle fibers already determined in the fetus, the postnatal growth of skeletal muscle is dominated by an increase in the size of the myofibers [39]. Hence, although some conversion between fast and slow muscle fibers occurs during the formation of primary and secondary myofibers, most of the conversion is taken place after birth. Skeletal muscle has the ability to change phenotype to adapt to developmental and environmental stimuli [40].

VA has long been considered an essential nutrient for animals. Recently, VA supplementation has been reported to increase the proportion of oxidized muscle fibers [23] and has the potential to improve meat quality of beef cattle [33]. In the present study, we observed that intramuscular injection of VA to sheep greatly enhanced the number of type I myofibers and reduced the number of type IIx myofibers in LD muscle. Meat color is commonly used by consumers to determine whether fresh meat is healthy or not, while myofiber type is one of the major contributors for meat color [41]. Our data suggested that the color of sheep meat may have changed. In cattle, Scapol et al. [42] found that intramuscular injection of VA into calves improved meat tenderness but did not affect meat color. Because meat color affected by various factors, including myoglobin [43], diet [44], and even the exercise it gets [45], whether VA injection affect the color of sheep meat need to be further explored.

Moreover, VA significantly improved mitochondrial biogenesis in LD muscle, including greatly increased mtDNA content, and *NRF1* and *TFAM* levels. Several studies have demonstrated that *NRF1* induced the transcription of mitochondrial respiratory chain genes and activated *TFAM* [46, 47], which promoted the mitochondrial DNA replication and transcription [48, 49]. This is consistent with more mitochondria being present in oxidative muscle fibers [50]. Moreover, mitochondria hold a central function in energy production and numerous metabolic processes [51]. Mitochondria synthesize ATP through the coupling of oxidative phosphorylation and respiration. This coupling needs the presence of mitochondrial membrane potential [52], which acts as an intermediate form of energy storage and can produce ATP in response to ATP synthase. Stable intracellular levels of ATP and mitochondrial membrane potential are necessary to maintain normal cellular function [53–55]. In our study, the enhancement of mitochondrial function in VA-treated sheep was demonstrated by increased succinate dehydrogenase (SDH) activity and the levels of UQCRC1 and ATP5A1 related to mitochondrial respiratory chain. Moreover, as a transcriptional co-activator, PGC-1α regulates genes involved in energy metabolism, and promotes the mitochondrial biogenesis. VA has been reported to promote mitochondrial production by activating PGC-1α [56–58]. Based on the previous results, we examined the PGC-1α expression in LD muscle of sheep and found that VA significantly upregulated PGC-1α mRNA and protein levels. What is more, we found that VA activated the p38 signaling pathway, an upstream signal of PGC-1α [59], which is consistent with previous findings [60].

Retinoic acid mediates most of the metabolic functions of VA. Retinoic acid in vivo is produced by retinol in the sequential action of retinol dehydrogenase and retinaldehyde dehydrogenase. Subsequently, retinoic acid binds to heterodimer of the retinoic acid receptor (RAR) and retinoic acid X receptor (RXR), and the complex binds to retinoic acid response elements (RAREs) in the regulatory regions of RA target genes, ultimately causing the transcription of the genes to be affected [61]. Consistent with the in vivo results, RA improved mitochondrial biogenesis and function, promoted the production of oxidative muscle fibers in sheep primary myoblasts. We further used si-PGC-1α demonstrated that this process was caused by RA through the regulation of PGC-1α, including that si-PGC-1α significantly attenuated RA-induced mtDNA content, *TFAM* and *NRF1* relative mRNA contents, MitoTracker staining intensity, UQCRC1 and ATP5A1 levels, and SDH activity. Thus, PGC-1α is a major factor contributing to the slow muscle fiber type [23, 62, 63]. Furthermore, p38 MAPK as an upstream signal of PGC-1α is activated by VA, but whether RA can alter muscle fiber type composition by activating the p38 MAPK-PGC-1α signaling pathway remains to be investigated. It has been reported that many cytokines stimulate PGC-1α via p38 MAPK leading to enhanced mitochondrial respiration and energy expenditure in muscle cells [28]. During muscle cell differentiation, retinoic acid activates p38 MAPK and promotes the ability of glucose uptake [60]. RA can induce *GADD34* mRNA to alter muscle fiber type through p38 MAPK signaling [64]. We so hypothesized that RA activated PGC-1α and altered muscle fiber type composition, partly due to the activation of p38 MAPK signaling. Our results showed that p38 MAPK inhibitor impaired RA-induced PGC-1α and MyHC I expression, but MyHC IIx levels were unaffected by p38 MAPK inhibitor, suggesting that RA increased the number of type I myofibers via p38 MAPK-PGC-1α signaling. Consistently, Shi et al. [65] also did not detect differences in phosphorylated p38 MAPK in fast muscle fibers. In contrast, Meissner et al. [66] reported that activation of p38 MAPK modulated MyHC IIx promoter activity and promoted *MyHC IIx* gene expression. Indeed, p38 MAPK contains 4 isoforms (α, β, γ, and δ), yet SB202190 selectively inhibited the activity of α and β isoforms. Whether the γ and δ isoforms play a role in muscle fiber type determination in our experiments needs to be further investigated. In addition, studies have shown that retinoic acid activates PPARs [67, 68], which are canonical activators of PGC-1α [69]. This suggests that retinoic acid may activate PGC-1α through PPARs. However, whether retinoic acid involved in myofiber conversion in sheep through PPARs-PGC-1α signaling pathway needs to be explored in depth.

## Conclusion

In conclusion, our results suggested that intramuscular VA injection improved mitochondrial biogenesis and function through PGC-1α signal, and increased the proportion of oxidized muscle fibers. Furthermore, we demonstrated that RA exerts these effects in part through the p38 MAPK pathway. More importantly, the results of this study provided new insights into the improvement of sheep meat quality and human’s living standards.

### Supplementary Information

**Additional file 1. **Protein marker and original gels of the Western blots in the manuscript.

## Data Availability

The datasets and materials are available from the corresponding author on reasonable request.

## Abbreviations

ATP5A1: ATP synthase F1 subunit alpha
LD: *Longissimus dorsi*
MDH: Malate dehydrogenase
MyHC: Myosin heavy chain
mtDNA: Mitochondrial DNA
NRF1: Nuclear respiratory factor 1
OCT compound: Optimal cutting temperature compound
PGC-1α: Peroxisome proliferator-activated receptor γ coactivator 1-α
p38 MAPK: p38 mitogen-activated protein kinases
RA: Retinoic acid
SDH: Succinate dehydrogenase
TEM: Transmission electron microscopy
TFAM: Mitochondrial transcription factor A
UQCRC1: Ubiquinol-cytochrome C reductase core protein 1
VA: Vitamin A
