# Supplementary material for: Vitamin A regulates mitochondrial biogenesis and function through p38 MAPK-PGC-1α signaling pathway and alters the muscle fiber composition of sheep
Source: J Anim Sci Biotechnol. 2024 Feb 4;15:18. doi: 10.1186/s40104-023-00968-4 (PMC10838450; doi:10.1186/s40104-023-00968-4)
Supplement: Supplementary file 1 — Additional file 1. Protein marker and original gels of the Western blots in the manuscript. [file 40104_2023_968_MOESM1_ESM.docx]

**Vitamin A regulates mitochondrial biogenesis and function through p38 MAPK-PGC-1α signaling pathway and alters the muscle fiber composition of sheep**

1. Protein markers (#MF212 (10-180 kDa) and #MF290-plus (10-310 kDa), purchased from Mei5 Biotechnology Co., Ltd. (Beijing, China)) we used were shown below:


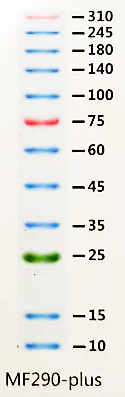

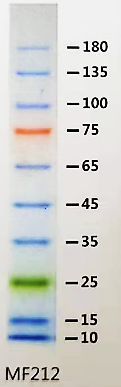


2. The original gels of the Western blots in manuscript. Protein Lanes in manuscript.

Fig. 1D. MHC Ⅰ


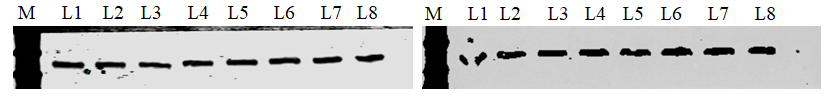


M: protein marker

L1-L2: MHC Ⅰ from control-treated lambs; L3-L4: MHC Ⅰ from Vitamin A-treated lambs

Fig. 1D. MHC Ⅱx


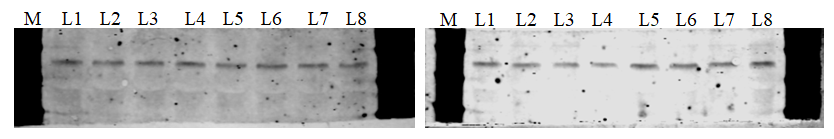


M: protein marker

L1-L2: MHC Ⅱx from control-treated lambs; L3-L4: MHC Ⅱx from Vitamin A-treated lambs

Fig. 1D. β-tubulin


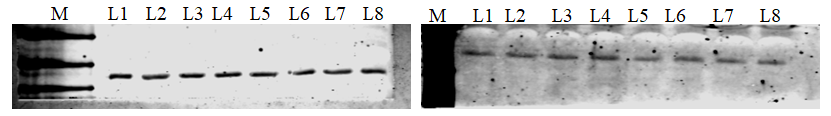


M: protein marker

L1-L2: β-tubulin from control-treated lambs; L3-L4: β-tubulin from Vitamin A-treated lambs

Fig. 1H. MHC Ⅰ


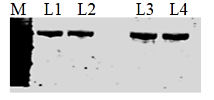


M: protein marker

L1-L2: MHC Ⅰ from control-treated lambs; L3-L4: MHC Ⅰ from Vitamin A-treated lambs

Fig. 1H. MHC Ⅱx


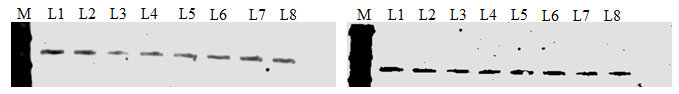


M: protein marker

L1-L2: MHC Ⅱx from control-treated lambs; L3-L4: MHC Ⅱx from Vitamin A-treated lambs

Fig. 1H. β-tubulin


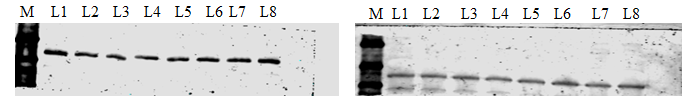


M: protein marker

L1-L2: β-tubulin from control-treated lambs; L3-L4: β-tubulin from Vitamin A-treated lambs

Fig. 2D. UQCRC1


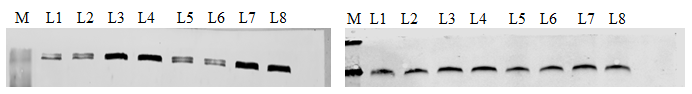


M: protein marker

L1-L2: UQCRC1 from control-treated lambs; L3-L4: UQCRC1 from Vitamin A-treated lambs

Fig. 2D. ATP5A1


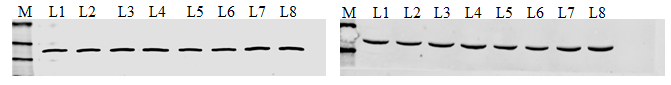


M: protein marker

L1-L2: ATP5A1 from control-treated lambs; L3-L4: ATP5A1 from Vitamin A-treated lambs

Fig. 2D. β-tubulin


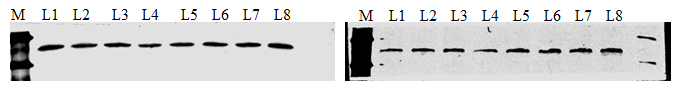


M: protein marker

L1-L2: β-tubulin from control-treated lambs; L3-L4: β-tubulin from Vitamin A-treated lambs

Fig. 2G. PGC-1α


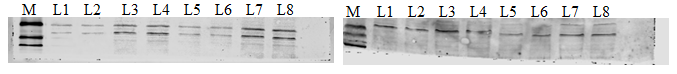


M: protein marker

L1-L2: PGC-1α from control-treated lambs; L3-L4: PGC-1α from Vitamin A-treated lambs

Fig. 2G. β-tubulin


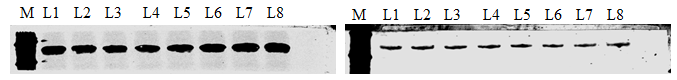


M: protein marker

L1-L2: β-tubulin from control-treated lambs; L3-L4: β-tubulin from Vitamin A-treated lambs

Fig. 2H. p38


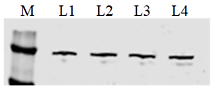


M: protein marker

L1-L2: p38 from control-treated lambs; L3-L4: p38 from Vitamin A-treated lambs

M: protein marker

Fig. 2H. p-p38


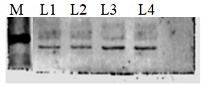


M: protein marker

L1-L2: p-p38 from control-treated lambs; L3-L4: p-p38 from Vitamin A-treated lambs

Fig. 2H. β-tubulin


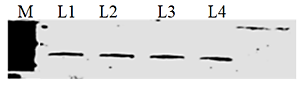


M: protein marker

L1-L2: β-tubulin from control-treated lambs; L3-L4: β-tubulin from Vitamin A-treated lambs

Fig. 3B. MHC Ⅰ


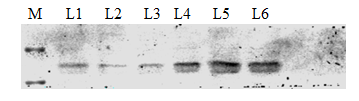


M: protein marker

L1-L3: MHC Ⅰ from control-treated myoblasts; L4-L6: MHC Ⅰ from RA-treated myoblasts

Fig. 3B. MHC Ⅱx


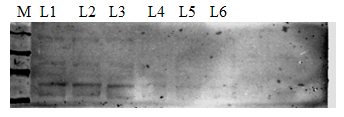


M: protein marker

L1-L3: MHC Ⅱx from control-treated myoblasts; L4-L6: MHC Ⅱx from RA-treated myoblasts

Fig. 3B. β-actin


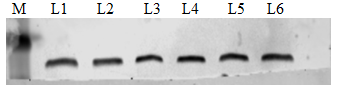


M: protein marker

L1-L3: β-actin from control-treated myoblasts; L4-L6: β-actin from RA-treated myoblasts

Fig. 3F. UQCRC1


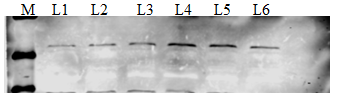


M: protein marker

L1-L3: UQCRC1 from control-treated myoblasts; L4-L6: UQCRC1 from RA-treated myoblasts

Fig. 3F. ATP5A1


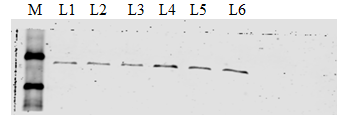


M: protein marker

L1-L3: ATP5A1 from control-treated myoblasts; L4-L6: ATP5A1 from RA-treated myoblasts

Fig. 3F. β-actin


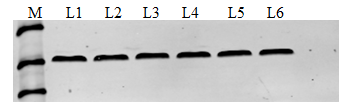


M: protein marker

L1-L3: β-actin from control-treated myoblasts; L4-L6: β-actin from RA-treated myoblasts

Fig. 4C. MHC Ⅰ


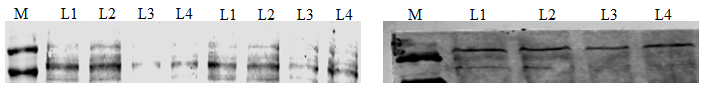


M: protein marker

L1: MHC Ⅰ from (si-NC)-treated myoblasts; L2: MHC Ⅰ from (siNC+RA)-treated myoblasts; L3: MHC Ⅰ from (si-PGC-1α)-treated myoblasts; L4: MHC Ⅰ from (si- PGC-1α+RA)-treated myoblasts.

Fig. 4C. MHC Ⅱx


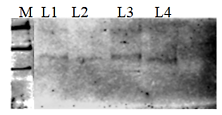


M: protein marker

L1: MHC Ⅱx from (si-NC)-treated myoblasts; L2: MHC Ⅱx from (siNC+RA)-treated myoblasts; L3: MHC Ⅱx from (si-PGC-1α)-treated myoblasts; L4: MHC Ⅱx from (si- PGC-1α+RA)-treated myoblasts;

Fig. 4C. β-actin


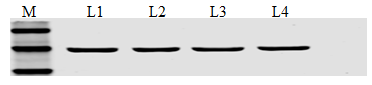


M: protein marker

L1: β-actin from (si-NC)-treated myoblasts; L2: β-actin from (siNC+RA)-treated myoblasts; L3: β-actin from (si-PGC-1α)-treated myoblasts; L4: β-actin from (si- PGC-1α+RA)-treated myoblasts;

Fig. 5F. UQCRC1


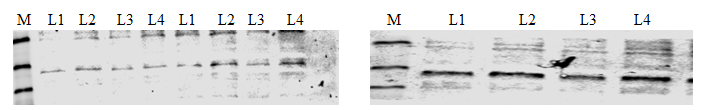


M: protein marker

L1: UQCRC1 from (si-NC)-treated myoblasts; L2: UQCRC1 from (siNC+RA)-treated myoblasts; L3: UQCRC1 from (si-PGC-1α)-treated myoblasts; L4: UQCRC1 from (si- PGC-1α+RA)-treated myoblasts;

Fig. 5F. ATP5A1


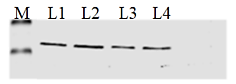


M: protein marker

L1: ATP5A1 from (si-NC)-treated myoblasts; L2: ATP5A1 from (siNC+RA)-treated myoblasts; L3: ATP5A1 from (si-PGC-1α)-treated myoblasts; L4: ATP5A1 from (si- PGC-1α+RA)-treated myoblasts;

Fig. 5F. β-actin


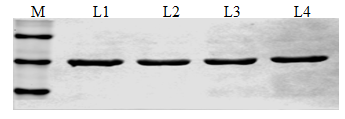


M: protein marker

L1: β-actin from (si-NC)-treated myoblasts; L2: β-actin from (siNC+RA)-treated myoblasts; L3: β-actin from (si-PGC-1α)-treated myoblasts; L4: β-actin from (si- PGC-1α+RA)-treated myoblasts;

Fig. 6C. PGC-1α


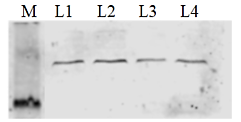


M: protein marker

L1: PGC-1α from (NC)-treated myoblasts; L2: PGC-1α from (NC+RA)-treated myoblasts; L3: PGC-1α from (p38 inhibitor)-treated myoblasts; L4: PGC-1α from (p38 inhibitor+RA)-treated myoblasts;

Fig. 6C. MHC Ⅰ


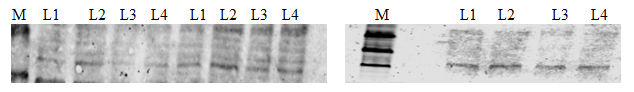


M: protein marker

L1: MHC Ⅰ from (NC)-treated myoblasts; L2: MHC Ⅰ from (NC+RA)-treated myoblasts; L3: MHC Ⅰ from (p38 inhibitor)-treated myoblasts; L4: MHC Ⅰ from (p38 inhibitor+RA)-treated myoblasts;

Fig. 6C. MHC Ⅱx


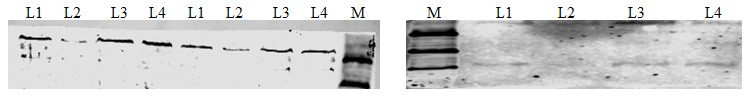


M: protein marker

L1: MHC Ⅱx from (NC)-treated myoblasts; L2: MHC Ⅱx from (NC+RA)-treated myoblasts; L3: MHC Ⅱx from (p38 inhibitor)-treated myoblasts; L4: MHC Ⅱx n from (p38 inhibitor+RA)-treated myoblasts;

Fig. 6C. β-actin


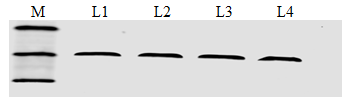


M: protein marker

L1: β-actin from (NC)-treated myoblasts; L2: β-actin from (NC+RA)-treated myoblasts; L3: β-actin from (p38 inhibitor)-treated myoblasts; L4: β-actin from (p38 inhibitor+RA)-treated myoblasts;
